# Study protocol and methods for Easing Pelvic Pain Interventions Clinical Research Program (EPPIC): a randomized clinical trial of brief, low-intensity, transdiagnostic cognitive behavioral therapy vs education/support for urologic chronic pelvic pain syndrome (UCPPS)

**DOI:** 10.1186/s13063-022-06554-9

**Published:** 2022-08-13

**Authors:** Jeffrey M. Lackner, James Jaccard, Brian M. Quigley, Tova S. Ablove, Teresa L. Danforth, Rebecca S. Firth, Gregory D. Gudleski, Susan S. Krasner, Christopher D. Radziwon, Alison M. Vargovich, J. Quentin Clemens, Bruce D. Naliboff

**Affiliations:** 1 Division of Behavioral Medicine, Department of Medicine, Jacobs School of Medicine, University at Buffalo, Buffalo, NY USA; 2 School of Social Work, New York University, New York, NY USA; 3 Department of Urology, University of Michigan, Ann Arbor, MI USA; 4 Department of Obstetrics and Gynecology, Jacobs School of Medicine, University at Buffalo, Buffalo, NY USA; 5 Department of Urology, Jacobs School of Medicine, University at Buffalo, Buffalo, NY USA; 6 G. Oppenheimer Center for Neurobiology of Stress and Resilience, Department of Psychiatry and Biobehavioral Sciences, UCLA, Los Angeles, CA USA

**Keywords:** Chronic pain, Randomized clinical trial, Transdiagnostic, Chronic prostatitis, Interstitial cystitis, Bladder pain syndrome, Self-management

## Abstract

**Background:**

Urologic chronic pelvic pain syndrome (UCPPS) encompasses several common, costly, diagnoses including interstitial cystitis/bladder pain syndrome and chronic prostatitis/chronic pelvic pain syndrome that are poorly understood and inadequately treated with conventional medical therapies. Behavioral strategies, recommended as a first-line treatment for managing symptoms, are largely inaccessible, time and labor intensive, and technically complex. The Easing Pelvic Pain Interventions Clinical Research Program (EPPIC) is a clinical trial examining the efficacy of low-intensity cognitive behavioral therapy (Minimal Contact CBT or MC-CBT) for UCPPS and its durability 3 and 6 months post treatment. Additional aims include characterizing the operative processes (e.g., cognitive distancing, context sensitivity, coping flexibility, repetitive negative thought) that drive MC-CBT-induced symptom relief and pre-treatment patient variables that moderate differential response.

**Methods:**

UCPPS patients (240) ages 18–70 years, any gender, ethnicity, and race, will be randomized to 4-session MC-CBT or a credible, non-specific education comparator (EDU) that controls for the generic effects from simply going to treatment. Efficacy assessments will be administered at pre-treatment, 2 weeks, and 3 and 6 months post treatment-week acute phase. A novel statistical approach applied to micro-analytic mediator assessment schedule will permit the specification of the most effective CBT component(s) that drive symptom relief.

**Discussion:**

Empirical validation of a low-intensity self-management therapy transdiagnostic in scope has the potential to improve the health of chronic pelvic pain patients refractory to medical therapies, reduce social and economic costs, conserve health care resources, as well as inform evidence-based practice guidelines. Identification of change mechanisms and moderators of treatment effects can provide proactive patient-treatment matching fundamental to goals of personalized medicine.

**Trial Registration:**

Clinicaltrials.gov NCT05127616. Registered on 9/19/21.

**Supplementary Information:**

The online version contains supplementary material available at 10.1186/s13063-022-06554-9.

## Background and rationale

Urologic chronic pelvic pain syndrome (UCPPS) represents a significant public health challenge for nearly 5 million Americans [1, 2]. Encompassing chronic prostatitis/chronic pelvic pain syndrome among men and interstitial cystitis (IC)/bladder pain syndrome among men and women, their cardinal symptom is chronic pain in the pelvic region often accompanied by urinary urgency and/or frequency. UCPPS symptoms lack a reliable biomarker and are not satisfactorily treated with conventional medical, dietary or rehabilitative treatments [3]. Further complicating treatment is the frequent co-occurrence of a cluster of 10 centrally-mediated pain conditions including, but not limited to, temporomandibular joint disorder (TMD), fibromyalgia (FM), chronic fatigue syndrome (CFS), benign headache, idiopathic low back pain, and irritable bowel syndrome (IBS) [4–6]. Their frequent co-aggregation at higher than chance rate and the growing public health impact of these conditions [7] led the US Congress and the NIH [8] to designate them as Chronic Overlapping Pain Conditions (COPC) [9]. When present, non-urological COPCs may interact with UCPPS to potentiate its onset [10], increase bladder sensitivity [11], reduce patient’s quality of life (QOL), complicate clinical-decision making, and compromise treatment response [12]. As with other COPCs, delay in diagnosis and treatment for UCPPS can have serious consequences including exacerbation of both organ-specific and body-wide physical symptoms. Recent research has shown [13] showing that the presence of more severe non-urological symptoms and more widespread pain are such strong predictors of urologic outcomes that they represent a possible marker of “centralized” [14, 15] somatic symptoms.

Beyond its symptom burden, UCPPS exacts economic costs [16, 17] conservatively estimated at $881.5 million in outpatient care for female sufferers alone. Broader chronic pain research [18] suggests that centralized pain disorders like UCPPS impose a “triple whammy” stressing the self-regulatory capacity [19–21] of those with (1) self-regulatory deficits who attempt to manage a (2) painful multi-symptom disorder with (3) impacts across multiple domains. Indeed, UCPPS patients suffer considerably throughout their lives from both UCPPS symptoms as well as associated psychological, social, and economic distress from social isolation [22], anxiety, depression [23] with and without pain-induced suicidal ideation [24], loss of work productivity, and reduced QOL [23, 25, 26].

In the absence of curative therapies, American Urological Association (AUA) Guidelines [27] recommend behavioral self-management strategies as a first line treatment for all UCPPS patients. Multiple small-scale RCTs have supported the efficacy of cognitive behavioral therapy (CBT) for UCPPS [28–31] and frequently co-aggregating pain conditions [32–35]. Despite its recognition as the “gold standard psychological treatment” for chronic pain disorders [36, 37], CBT’s public health impact is limited because it does not reach many in need [38, 39]. A major obstacle is the dominance of a treatment delivery model [40] characterized by 5 interconnected features: CBT is typically delivered (1) individually (2) by specialized, costlier providers (of which there are relatively few), (3) who administer relatively complex [41] treatments teaching high-level skills [42, 43], (4) over extended periods (16–20 sessions) (5) in outpatient settings in affluent urban centers [44]. These factors restrict CBT’s scalability and access to many of our most vulnerable (e.g., rural, minority, lower income). This situation is particularly challenging for UCPPS patients because non-urologic comorbidities co-aggregate particularly those in the bladder-gut axis (IBS, GERD) [11]. The notion that a typical UCPPS patient would undergo separate behavioral regimens for pelvic pain and each comorbidity (e.g., IBS, anxiety, depression, low back pain) is impractical, costly, and beyond the skill of providers, most of whom receive limited training in single diagnosis protocols [45, 46] even i*f *there were sufficient numbers to support demand. It is not surprising that “feasibility” concerns have tempered AUA’s endorsement of behavioral self-management in its practice guidelines [27].

One way of increasing efficiency of treatment delivery involves working around the structural barriers (e.g., time constraints, inaccessibility, transportation problems cost, etc.) that make it extremely difficult or impossible to attend regular therapy sessions. In low-intensity or *minimal contact* (MC) treatments [47] for example, the patient receives a limited number of face-to-face clinic sessions with most skills learned independently using home lessons reinforced through self-study materials. Beyond an efficacy profile equivalent to high intensity CBT for centralized pain disorders [48], MC-CBT advantages include greater patient satisfaction [48], good homework compliance (68%) [48], lower dropout (9% [48] vs 13–26% for traditional CBT [49, 50]), rapid onset of action [51], and reduced cost ($348 [52] vs. $715 [53] for traditional CBT).

Another way of increasing treatment delivery efficiency is to develop more parsimonious transdiagnostic or “across disorder” interventions. Because transdiagnostic interventions target shared processes underlying mechanistically related disorders, they emphasize symptom-driving commonalties across different disorders rather than their relatively superficial symptomatic or diagnostic differences. Emphasizing a common set of therapeutic procedures effective for a class of mechanistically similar disorders could improve real-world applicability of treatment protocols by reducing their complexity, cost, and training demands of community-based practitioners who are overwhelmed with the number of single disorder treatment protocols validated through RCTs. In the case of the 10 COPCs alone, RCTs have generated over 170 disorder-specific behavioral pain protocols since 1988 [54]. What has historically qualified as a source of scientific innovation (i.e., development of empirically validated treatments) is increasingly criticized as “accomplish[ing] only a series of incremental advances,” [55] being time-consuming, costly, and inefficient [56] leading to front-line clinicians suffering from “too many empirically supported treatments.” ([57] p. 58) The result is a widening science-to-service gap [58] that more accessible but not necessarily safer nor more effective pharmacological options increasingly fill [59] at a time when the public shows a threefold preference for behavioral treatments vs medications. Because transdiagnostic treatments target well-defined clusters of multimorbid disorders they allow clinician to treat simultaneously multiple physical *and* emotional symptoms, potentially increasing the efficacy, breadth, and efficiency of self-management treatments [60]. Targeting common core processes may relieve comorbid symptoms (e.g., fatigue, anxiety) that interact adversely with the principal complaint (pain) but fall through the diagnostic cracks or are altogether overlooked due to the circumscribed scope and expertise of provider [61]. Low-intensity transdiagnostic-based behavioral pain treatments can reduce cost inefficiencies of high-intensity behavioral pain treatments and are particularly well suited to settings (e.g., rural clinics) where providers serve a diagnostically heterogeneous population with low base rates for specific diagnoses within, for example, the COPC phenotype.

Consistent with this approach, we have found that a low-intensity, transdiagnostic CBT protocol targeting one COPC (IBS) had a particularly strong impact on the severity of multiple co-occurring COPCs, including UCPPS even though they were *not* explicitly targeted [62]. Among *non-targeted* COPCs, effect sizes (ES, Cohen’s *d*) [63] for an average reduction in pain severity from pre to post treatment ranged from a small effect for fibromyalgia (*d* = .24), to moderate effects for idiopathic low back pain (*d* = .45) and TMD (*d* = .51), to very large effects particularly for disorders of UCPPS (*d* =1.03). To put these findings in context, the effect for transdiagnostic CBT featured in the EPPIC yielded a pooled ES (*d* = .55) for relief of *non-targeted* pain severity comparable, if not greater, than the ES (~.20) [64] for targeted pain via traditional single-disorder behavioral pain treatments [65, 66]. These data lend preliminary support to the scientific premise that a transdiagnostically designed treatment of one centralized pain disorder within the sub-class of COPCs provides significant relief across other mechanistically similar, frequently co-occurring COPCs, including UCPPS.

### Trial design and participant timeline

**Fig. 1:**
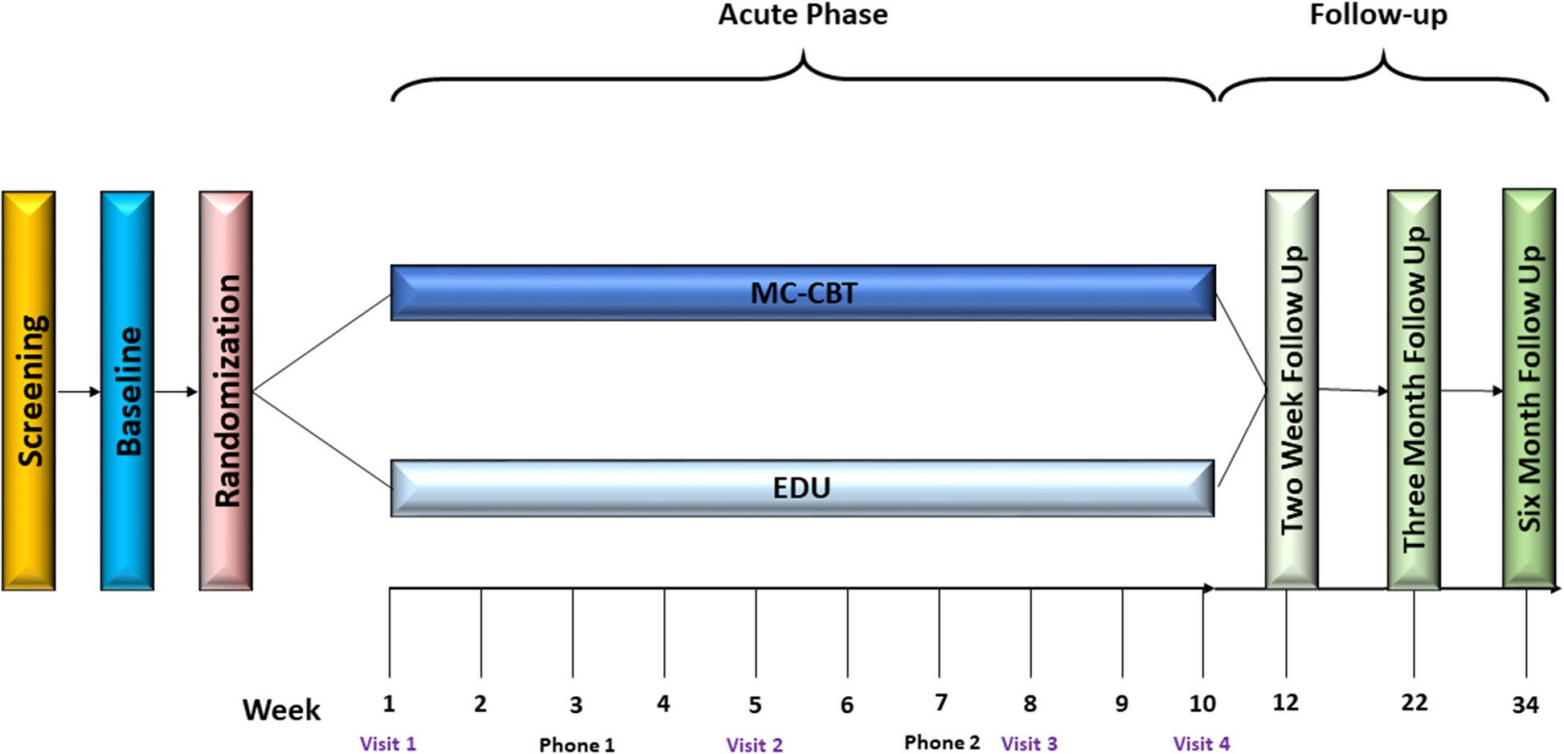
EPPIC study design and patient flow

EPPIC is a parallel-group, randomized, controlled trial with equal allocation to two arms without stratification. Arms include low-intensity versions of CBT and education/support (EDU) condition for UCCPS (see Fig. 1). Individuals will be phone screened for eligibility before a diagnosis (IC/BPS, CP/CPS) is formally confirmed, enrolled, and randomized to either CBT or EDU. Following pretreatment assessment and a two-week baseline period, a 10-week acute period will begin during which participants in both conditions will receive four in-person clinic visits on weeks 1, 5, 8, and 10 and brief phone follow-up on weeks 3 and 7 (see Fig. 1 and Table 3). Phone contact is designed to troubleshoot around any problems completing assigned home exercises (CBT) and to provide support and clarify understanding of education materials (EDU). Post treatment assessment will be conducted at 2 weeks, 3 months, and 6 months post treatment.

### Objectives and hypotheses

*Aim 1 (primary):* Evaluate the efficacy of MC-CBT for UCPPS as compared to EDU in relieving pelvic pain and related symptoms using key outcomes deemed important to stakeholders including patients, clinicians, researchers, payers, and policy makers.

- *Hypothesis 1a:* Patients randomized to MC-CBT will show greater global UCPPS symptom improvement on the primary endpoint (Clinical Global Improvement, CGI) compared to those randomized to EDU.
- *Hypothesis 1b:* MC-CBT will be superior to EDU on key secondary endpoints including the severity of pelvic pain and urinary symptoms, health-related quality of life, and functional impairment at post treatment.

*Aim 2:* Determine the durability of MC-CBT relative to EDU at 3 and 6 months.

- *Hypothesis 2:* Patients randomized to MC-CBT will show greater global UCPPS symptom improvement on the primary endpoint (CGI) compared to those randomized to EDU at 3 and 6 months.

*Aim 3:* Identify theoretically-informed and empirically-grounded mechanisms that explain how MC-CBT works and for whom it is more effective relative to EDU for the purpose of optimizing treatment effects.

- *Hypothesis 3a:* After mapping each MC-CBT procedure onto a specific, presumed mediator underlying symptom improvement, we (a) formally test if MC-CBT vs. EDU does, in fact, affect each change mechanism, and (b) determine the strength of the effect of each mechanism on UCPPS symptom improvement (CGI) and address the relative importance of different mechanisms in predicting CGI. By virtue of MC-CBT’s unique technical composition, cognitive processes (e.g., context sensitivity) will have mechanistic specificity that has not characterized pain-specific cognitions like pain catastrophizing [67].
- *Hypothesis 3b:* Because the ability to self-manage symptoms relies on both trait and state components of one’s self-regulatory capacity, trait self-regulation will moderate the impact of learning-based MC-CBT vs EDU on UCPPS symptom improvement at both post treatment and follow-ups.

## Method

The Easing Pelvic Pain Interventions Clinical Research Program (EPPIC) trial is funded by the NIDDK under the R01 mechanism and registered on clinicaltrials.gov. All procedures described below have been approved by the University at Buffalo Institutional Review Board. At a minimum, this protocol comports with SPIRIT reporting guidance [68] (see supplemental materials for SPIRIT checklist).

### Study setting

The clinical and administrative activities of EPPIC will take place in the clinic offices of the University at Buffalo’s Behavioral Medicine Division located at the Erie County Medical Center campus, an affiliated hospital of the Jacobs School of Medicine at the University at Buffalo. As a multi-site, EPPIC benefits from collaborations among experienced clinical scientists with subject matter expertise in biostatistics (Dr. Jaccard, NYU), UCPPS (Dr. Clemens, Michigan), and assessment (Dr. Naliboff, UCLA).

### Participants and eligibility criteria

Planned enrollment is 240 adults between ages 18 and 70 (inclusive) of any gender, race, or ethnicity who have been formally assigned a diagnosis of IC/BPS or CP/CPPS (confirmed by a board-certified study urologist or urogynecologist) with clinically significant pelvic pain present for at least 6 months. Table 1 lists the inclusion and exclusion (urologic and general) criteria with corresponding rationales.

**Table 1:** Participant inclusion and exclusion criteria

	Rationale
**Inclusion criteria**	
Ages 18–70 years inclusive	Matches developmental correlates of treatments and outcome domains
Males and females of any race, ethnicity, and gender	Includes the full range of individuals diagnosed with UCPPSs
Formal diagnosis of IC/BPS or CP/CPPS (confirmed by urologist or urogynecologist)	Optimizes construct validity of disorders of interest
Pelvic pain (including pressure or discomfort) of at least 6 months duration	Establish that pelvic pain is chronic, not acute
Pelvic pain intensity of at least moderate severity (defined as 3 or greater on a 0–10 scale and limits participant’s life or work-related activities) over the past 3 months	Establish that UCPPS symptoms are sufficiently clinically significant to detect improvement
Not taking medications and/or willing to refrain from starting new medications until after the initial 2-week baseline period ends unless medically necessary	Stabilization of baseline symptoms and confounding of treatment effects
**Urologic exclusion criteria**	
Presence of a neurological condition (e.g., multiple sclerosis Parkinson’s disease, paraplegia) affecting the bladder	Urological symptoms due to, or possibly result of, a specific disease condition that may require different treatment
Presence of a symptomatic urethral stricture (males only)	Urological symptom due to, or possibly result of, a specific disease condition
History of cystitis caused by tuberculosis or radiation or chemotherapies	Urological symptom due to, or possibly result of, a specific disease condition
Previous diagnosis and treatment for a pelvic-related malignancy (e.g., colon, bladder, prostate, ovarian, endometrial, uterine, testicular, penile, cervical, vaginal, or rectal cancer)	Condition/circumstance might confound treatment effects or interpretation of data
**General exclusion criteria**	
Existing medical condition(s) whose nature or severity could adversely influence the conduct of the clinical trial or compromise volunteer safety	Condition/circumstance might confound treatment effects or interpretation of data or compromise safety
Gross cognitive impairment, deafness, blindness, severe vision or hearing problems	Condition may make it difficult to attend sessions or complete home assignments
Presence of a major psychiatric disorder that would impede conduct of the clinical study [major depression with a high risk of suicidal behavior (i.e., intent or plan); current or recent (within the past 3 months) history of alcohol or substance abuse/dependence; a lifetime history of schizophrenia or schizoaffective disorder; or an organic mental disorder]	Condition might confound treatment effects or interpretation of data or compromise safety
Current involvement in psychotherapy directed specifically toward relief of urological symptoms	Possible bias due to exposure to experimental procedures
Unable to read or fluently speak English	Condition would make it difficult for fully informed consent or to participate in study

### Recruitment

We will adopt a two-pronged recruitment approach that emphasizes direct and indirect methods in an effort to optimize a sample representative of the range of patients with UCPPS. Indirect methods are aimed at enlisting support (e.g., referrals, generating positive word of mouth, building brand identity of the EPPIC) through an established network of “gatekeeper” physicians (e.g., primary, urologists and urogynecologists, OB/GYN), physical therapists, and other health care professionals who are in a position to engage participants in the EPPIC. Direct methods include efforts to promote EPPIC directly to patients in a wide variety of community locations including those frequented by patients with urologic conditions (urologists, urogynecologists, physical therapy offices, pharmacies, etc.) as well as at other community locales (coffee shops, places of worship, community centers, gyms), health fairs, and through local (e.g., newspapers, radio) media, social media (e.g., Facebook), study website (http://ubeppic.com/). We have also partnered with the Interstitial Cystitis Association in an effort to publicize the proposed trial through its clinical trial registry.

### Trial interventions

#### Minimal-contact CBT

MC-CBT involves four 50–60-min individual clinic sessions delivered over a 10-week acute phase. While descriptive content is tailored to UCPPS, the protocol synthesizes [69] evidence-based CBT strategies into 4 modules targeting core transdiagnostic vulnerability factors [70–73] reflecting a rigid cognitive style expressed as discrete perceptual biases [74]. These include (a) a tendency toward self-immersive, abstract, and repetitive negative thought (RNT) [70] manifested in (b) the inclination to overestimate the probability of negative events (threat expectancy bias) [75–78]; (c) the tendency to inflate their costs or consequences when they occur (threat interpretative bias) [79–81]; (d) extreme negative self-schemas [82] (i.e., dysfunctional misconceptions or core beliefs like perfectionism [83–85]); and (e) a rigid, non-discriminative coping style characterized by an overreliance on control-oriented, problem-focused strategies deployed regardless of situational demands (e.g., controllability) [86–90]. Technical components include “real time” self-monitoring to generate a functional analysis of symptoms, their triggers, and responses across multiple domains (cognitive, emotional, somatic, behavioral), diaphragmatic breathing to reduce arousal and enhance personal control, worry control (e.g., evidence-based logic, decatastrophizing) to correct maladaptive information processing style, flexible problem solving, and relapse prevention skills to maintain gains after treatment discontinuation. MC-CBT content is introduced sequentially and reinforced through the provision of a workbook [91] with home exercises designed to facilitate skills acquisition. MC-CBT components and corresponding transdiagnostic processes are based on prior research with transdiagnostically designed CBT [47, 48] and refined through stakeholder involvement (e.g., patients, physicians, physcians assistants, and therapists) for the EPPIC. Contents addressed in of each week of treatment are presented in Table 2.

**Table 2:** Content topics and transdiagnostic processes of MC-CBT and content topics of education/support by treatment session

Week	Schedule	MC-CBT content	MC-CBT transdiagnostic process	EDU content
1	Clinic visit 1	Treatment orientation and rationale; Acute vs chronic pain; Central sensitization; Self-monitoring (functional analysis); Introduction to relaxation	Perceived control over aversive internal states; Self-distancing (“fly on the wall”); Perspective broadening; Negative reactivity	Treatment orientation and rationale Education about Chronic Pelvic Pain (CPP) Emphasize value of emotional support Acute vs chronic pelvic pain, Symptom monitoring
2	Self-directed (no contact)	Relaxation training; Real-time self-monitoring (functional analysis)	Perceived control over aversive internal states; Self-distancing (“fly on the wall”); Perspective broadening;	Reflective writing narrative (RWN) about CPP and its personal impacts
3	Self-directed (phone check-in 1)	Portable relaxation training; Real-time self-monitoring (functional analysis)	Perceived control over aversive internal states; Self-distancing (“fly on the wall”); Perspective broadening	Understanding CPP; Review RWN Central sensitivity and CPP
4	Self-directed (no contact)	Introduction to cognitive flexibility model	Perceived control; Maladaptive worry/rumination; Repetitive negative thought (RNT)	Symptom triggers (emotions, relationships, family, work, etc.) of CPP
5	Clinic visit 2	Prediction testing; Evidence-based logic	RNT: Expectancy bias/prediction error (“What if?”)	The role of stress and CPP Review personalized Stress Profile
6	Self-directed (no contact)	Decatastrophizing	RNT: Interpretative bias (“If only…”)	Tracking dietary and physical activity triggers
7	Self-directed (phone check-in 2)	Applying evidence-based logic vs decatastrophizing skills to meet situational demands	RNT: Expectancy bias/Interpretative bias; Context sensitivity	Review activity and dietary triggers Pain and the stress response
8	Clinic visit 3	Flexible problem-solving training	Coping flexibility; Context sensitivity	Diet, activity and CPP
9	Self-directed (no contact)	Flexible problem solving Schema (core beliefs) modification	Coping flexibility; Context sensitivity; Negative self-schema (e.g., perfectionism)	Integrate knowledge learned about personalized triggers with biopsychosocial model
10	Clinic visit 4	Maintenance/relapse prevention	Maintenance	Review biopsychosocial model of CPP

#### UCPPS Education

EDU is delivered in four 50–60-min individual clinic sessions and structured around information dissemination, support, and reflection. Content includes information about chronic pelvic pain and its clinical features, epidemiology, diagnostic criteria, medical tests, and treatment options as well as the role of stress, diet, and physical activity (Table 2). To control for home exercises of MC-CBT, subjects receive a science-based pelvic pain education book [92] that emphasizes the therapeutic value of knowledge ("the more you know about pain, the better off you'll be"), track UCPPS symptoms (but not corresponding thoughts, behaviors, and emotions), and complete a stress profile [93] without prescriptive behavior changes overlapping with CBT.

#### Explanation of choice of comparators

EPPIC’s UCPPS Education (EDU) condition conforms to the best practice [67, 94] of a nonspecific comparator structurally equivalent to MC-CBT (credibility, time, attention, therapist training, etc.). By comparing CBT to a non-specific education condition, we will be able to discern whether treatment effects reflect the benefits of specific CBT’s technical components above and beyond the generic effects of simply going to treatment that comes from feeling listened to and receiving support, mobilizing positive expectancy for improvement, establishing a therapeutic relationship around working toward shared goals with a trusted and knowledgeable clinician.

### Sequence generation, concealment, and implementation

Simple randomization into one of the two equal-probability conditions in a 1:1 ratio on a continuous basis as participants qualify for allocation will be performed by the study coordinator who has no patient care responsibilities as a safeguard against selection bias. In addition, treatment assignment will be conducted using Randomization Module in Research Electronic Data Capture (REDCap) software [95], which *generates* random and unpredictable sequence of assignments. The details of its computer-generated randomization algorithm are unknown to members of the EPPIC research team. Allocation sequence *concealment* from study personnel is achieved because REDCap conceals the next treatment assignment from being known. In other words, neither participants nor members of the research team are aware of the generated sequence until (and only for) the participant is assigned to his/her respective condition. Allocation sequence is generated by a computer independent of research coordinator who *implements* the assignment. Because the two treatments have identical dosages (4 sessions), the condition to which the participant is assigned is not revealed to him/her until session 1 of the acute phase, further minimizing selection bias.

### Concomitant care policy

To optimize the external validity of study findings and expedite accrual, participants of both conditions will be permitted to continue with or modify the treatment they were engaged through the acute phase with exception of ongoing pelvic pain-targeted psychological therapy which is disallowed for allocation. To strike a balance between methodological (rigor) and ethical (safety) concerns, patients will be encouraged to maintain concomitant care use during the baseline period for the purpose of establishing a stable reference for gauging treatment effects. It is our policy that an outright requirement of maintaining a stable dose through the baseline period exposes the participant to an unjustified level of risk should s/he experience a serious health event for which a change in medications or other therapies is medically necessary for and represents a higher order consideration than internal validity. Because the likelihood of serious health events is expected to be low for this population, encouraging patients to maintain stable doses through the baseline period does not diminish rigor and may actually increase participant engagement that optimizes overall internal validity. We also believe that our approach results in a more representative sample that includes participants with medical comorbidities for which medications are often prescribed. Beyond ethical issues, stabilizing concomitant therapies during the acute treatment phase may distort the therapeutic benefit of a self-management treatment for participants who learn to control symptoms for which they no longer require pharmacological, rehabilitative, or dietary interventions reported at baseline. A stronger methodological approach is to assess concomitant health care (e.g., dosage, type) which we will capture via self-report, factor into statistical analyses, and present in the final report.

### Blinding

A board-certified urologist or urogynecologist will confirm medical eligibility using formal diagnostic criteria for IC/BPS or CP/CPPS at baseline for all patients and function as independent evaluators (“blind” to treatment assignment) of symptom improvement at immediate (week 12), 3 months and 6 months follow-ups. Participants will be unaware of study hypotheses and blind to treatment assignment through the pretreatment baseline period. The methodological criterion of blinding participants to assigned treatments is inapplicable to behavioral interventions [96]. To the extent that blinding controls for differential expectations and consequent demand characteristics they may generate, we will adopt the established, surrogate practice [96, 97] of having participants rate the credibility and expectancy of improvement of the treatment to which they were assigned using the Credibility/Expectancy Scale [98] at the end of session 1 (week 1). Statisticians will be blinded to treatment allocation during the study by analyzing deidentified data until data is unlocked [99].

### Retention and compliance

To optimize session patient retention and compliance, we will provide reminders via their preferred method of text, telephone call, or email within 1 business day of the scheduled appointment. We will provide an honorarium for travel, time, and convenience for assessments: Initial assessment ($25); interim ($25); and 2 weeks ($50), 3 months ($50), and 6 months ($50) follow-ups. Patients who complete 75% of sessions will be regarded a priori as having received a clinically thorough regimen of treatment to which they were assigned (e.g., compliers). Additional strategies are codified in a retention plan that covers areas such as staff training for initial contact, early detection of patient behaviors that may “red flag” correctable adherence problems (e.g., work conflict), formal training in rapport building, and positive staff-patient communication skills, creating a welcoming and respectful environment for participants, and educating participants about their role as participants and the role of participation incentives. Other procedures to minimize attrition and non-adherence include engendering trust, maintaining relevance to clients’ needs, establishing routine while maintaining a degree of flexibility in scheduling to maintain engagement in both treatment and assessment phases of a study, therapist techniques for “rolling with resistance”, and other brief motivational enhancement strategies that are uniformly applied across conditions and therefore do not represent a source of bias. Secondary indices of compliance include the number of no-shows (failing to show without contacting office), and canceled appointments without rescheduling. Compliance with weekly home exercises will be measured using a 6-point clinician rating scale ranging from 1 (0%) to 6 (>100%) [48, 100]. For participants who prematurely discontinue treatment (dropouts), we will identify self-reported reasons for withdrawal and record them. Reason(s) for dropout will be coded using five categories: logistical (e.g., childcare coverage), treatment/program related (e.g., participant prefers different treatment, stopped because they felt better, it failed to meet their needs, not the right time to engage in treatment); influence of others (e.g., treating doctor advised against continuing); study staff reasons (e.g., eligibility failure due to non-disclosure of information that would have rendered patient ineligible); miscellaneous (e.g., death). Participants who discontinue treatment will be encouraged to complete follow-up assessments in an effort to optimize intent to treat (ITT) analyses.

### Treatment fidelity

To optimize the quality of and adherence to CBT and EDU, therapists will receive extensive training in the components of each treatment under expert supervision before being assigned to study patients. Delivery will be optimized by treatment manuals that provide detailed session-by-session guidance to standardize intervention across therapists; the completion of checklists for session protocols after each session; and regularly scheduled supervision with senior clinicians. Sessions will be audio taped, 25% of which will be randomly selected per patient and rated for protocol adherence by an independent rater unassigned to treatment delivery.

### Data collection and management

#### Primary outcome measure

The patient version of the Clinical Global Impressions - Improvement Scale (CGI-I) is a 7-point centered scale that integrates symptom severity and improvement over time as the primary outcome measure. Specific UCPPS-based anchors points for rating the CGI will be appropriately added as is the convention for other multi-symptom disease states [101] including chronic pelvic pain [102]. Global ratings of UCPPS symptom improvement yield a measure of overall multi-symptom benefit from treatment and is a core outcome domain in pain RCTs [103]. Those who score 2 (much improved) or better at follow-up qualify as categorical responders. Patients will complete the CGI at the three follow-up assessments and, for process analyses (Aim 3), weeks 3, 5, 7, 8, and 10 of the acute phase. The clinician version of the CGI will be completed at follow-up by “blind” MDs masked to treatment assignment to minimize bias and establish the validity of the patient version [48].

#### Secondary outcome measures

Secondary outcome measures include pelvic pain, urinary symptoms, pain interference, emotional distress, quality of life, and patient satisfaction using measures with confirmed psychometric properties (see Table 3). Severity of urinary symptoms and pelvic pain will be assessed using factorially derived items from the Genitourinary Pain Index (GUPI) [104] and the Interstitial Cystitis Symptom (ICSI) and Problem Indices (ICPI) [105]. Pain interference will be assessed using the PROMIS - Pain Interference scale (PPI SF-6a) [106], a 6-item instrument of the consequences of pain on relevant aspects of one’s life, including social, cognitive, emotional, physical, and recreational activities. Emotional distress will be measured using the Brief Symptom Inventory-18 (BSI-18) [107] which measures the level of distress across three dimensions (i.e., anxiety, somatization, and depression). The Client Satisfaction Questionnaire (CSQ) [108], an 8-item instrument measuring patient satisfaction with treatment, will assess the quality of care at immediate post treatment. Quality of life and co-morbid COPCs will be measured with the 12-item version of the SF-36 Health Status Questionniare [109] and 41-item Complex Multi-Symptom Inventory (CMSI) [110] respectively. UCPPS symptom measures (GUPI, ICPI, ICSI) will be assessed at baseline, interim (weeks 3, 5, 7, 8, and 10 of acute phase), and at all follow-up visits. As non-urological secondary outcomes, the BSI-18, CSQ, CMSI, SF-12, and PPI SF-6a will be measured at baseline and post treatment.

**Table 3:** Enrollment, intervention, and assessment schedule for primary outcomes, mediators, and predictors

	Screening	BL	Acute phase						Follow-up		
Timepoint (week)	−2	0	1	3	5	7	8	10	12	22	34
**Enrollment:**											
Eligibility confirmation	x										
Informed consent	x										
Allocation		x									
**Interventions:**											
Minimal-contact CBT (MC-CBT)			**X**	T	**X**	T	**X**	**X**			
UCPPS Education			**X**	T	**X**	T	**X**	**X**			
**Assessments:**											
*Primary outcome*											
Global symptom improvement (CGI-I)				x		x	x	x	x	x	x
*Secondary outcomes*											
Emotional distress (BSI-18)		x							x	x	x
Patient satisfaction (CSQ)									x		
Comorbid COPCs (CMSI)		x							x	x	x
Pelvic pain severity (GUPI)		x		x		x	x	x	x	x	x
Urinary problem severity (ICPI)		x		x		x	x	x	x	x	x
Urinary symptom severity (ICSI)		x		x		x	x	x	x	x	x
Pain interference (PPI SF-6a)		x							x	x	x
Quality of life (SF-12 v2)		x							x	x	x
*Mediators*											
Perspective broadening (CERQ-PB)		x		x					x	x	x
Self-distancing (EQ-D)		x		x					x	x	x
Perceived control (MAIA-2)		x		x					x	x	x
Repetitive thinking (PTQ)		x				x			x	x	x
Context sensitivity (CSI)		x					x		x	x	x
Coping flexibility (CFS-R)		x						x	x	x	x
*Prescriptive predictors (moderators)*											
Trait self-regulation (BRIEF-A)		x									
*Prognostic predictors*											
Comorbidities: medical and psychiatric		x							x	x	x
Concomitant treatments		x							x	x	x
Demographics, chronicity, etc.		x									

*BL* baseline, *CGI-I* Clinical Global Impression-Improvement, *BSI-18* Brief Symptom Inventory-18, *CSQ* Client Satisfaction Questionnaire, *CMSI* Complex Medical Symptoms Inventory, *GUPI* Genitourinary Pain Index, *ICPI* Interstitial Cystitis Problem Index, *ICSI* Interstitial Cystitis Symptom Index, *PPI SF-6a* PROMIS - Pain Interference SF-6a, *SF-12 v2* Short Form-12 Health Survey v2, *CERQ-PB* Cognitive Emotion Regulation Questionnaire-Perspective Broadening, *EQ-D* Experiences Questionnaire-Decentering, *MAIA-2* Multidimensional Assessment of Interoceptive Awareness-2 (Noticing, Non-Distracting, Not-Worrying and Attention Regulation scales), *PTQ* Perseverative Thinking Questionnaire, *CSI* Context Sensitivity Index, *CFS-R* Coping Flexibility Scale-Revised, *BRIEF-A* Behavior Rating Inventory of Executive Functioning-Adult. Bolded Capped X = clinic session (intervention); T = telephone check in (intervention)

#### Mediators

The primary mediators are designed to tap into aspects of a rigid cognitive style central to a transdiagnostic conceptual model of centralized pain states such as UCPPS [111]. Mechanistic outcomes believed to drive CBT include context sensitivity, coping flexibility, repetitive thinking, self-distancing/perceived control, all of which are targeted by a different treatment module (see Table 2). All mediators will be assessed at baseline and follow-ups. The timing of additional mediator assessment is calibrated to the weeks when the corresponding skill believed to induce respective cognitive change is introduced and practiced. For example, because flexible problem solving is believed to improve symptom improvement by increasing sensitivity to contextual cues that promote coping (i.e., context sensitivity), the Context Sensitivity Questionnaire (CSI) [112] is administered at week 8 after the flexible problem-solving module is introduced and practiced. This approach differs from other assessment schedules of mechanistic studies when all putative mediators are assessed across different interim assessment periods (e.g., weeks 3, 5, 8) without regard to the mechanistic specificity of each strategy within a protocol [67]. By the same token, self-distancing, measured with the 11-item Experiences Questionnaire - Decentering (EQ-D) [113] and the Perspective Broadening scale of the Cognitive Emotion Regulation Questionnaire (CERQ-PB) [114] will be assessed at week 3 as will 4 scales (i.e., Noticing, Non-Distracting, Not-Worrying, Attention Regulation) of the Multidimensional Assessment of Interoceptive Awareness - 2 (MAIA-2) [115] measuring perceived control over aversive somatic sensations. Repetitive thinking will be assessed at week 7 and at all follow-ups with the Perseverative Thinking Questionnaire (PTQ) [116]. The Coping Flexibility Scale - Revised (CFS-R) [117], a 12-item instrument designed to measure discontinuation of ineffective coping strategies; re-coping, and meta-coping, will be assessed at baseline, week 10 and all follow-ups. Non-specific mediators common to both EDU and CBT include treatment expectancy (Credibility/Expectancy Questionnaire) [98] which will be assessed at the end of session 1 (week 1), while therapeutic alliance (Working Alliance Inventory) [118] which be assessed at weeks 1, 3, 5, 8, and 10.

#### Predictors and covariates

The primary theoretical-based predictor with prescriptive value (i.e., moderator) is trait self-regulation [119] which we will assess using the 75-item, self-report Behavior Rating Inventory of Executive Functioning - Adult (BRIEF-A) [120]. The BRIEF-A assesses habitual propensity with self-regulating or executive function within the context of everyday life. It is reliably associated with mental health, health behaviors, and physical health parameters [119]. Demographic variables, medication use, and disease characteristics (e.g., symptom severity, chronicity, treatment history, comorbidities) will be explored as general, non-specific predictors with prognostic value. Prescriptive and prognostic variables will be assessed at baseline.

#### Data collection and management

Sources of research material will include clinical data from structured interviews, self-report measures, physician assessments, and audio-recorded treatment sessions which will be used to establish therapist fidelity to treatment protocols. Clinical data will be captured using research electronic data capture (REDCap) software [95]. REDCap is a secure, Health Insurance Portability and Accountability Act compliant, web-based application designed to support data capture for research studies. Data will be collected for research purposes only and only with consent of the study volunteer released to a designated recipient (e.g., physician). Original hard copy source documents will be kept in study binders in locked cabinets or electronically stored on a secure server that will be encrypted and password protected. Data for analysis will be stored on a study-specific, password-protected database using subject numbers without personal identifiers. All data are backed up on external servers on a daily basis to a central secure data serve at UB. Access is password protected at multiple levels and no member of the EPPIC team apart from those with data management responsibility will have access to these passwords. Digital records of sessions will be stored in a secure, password-protected folder on the UB server. For all data, separate, an encrypted file linking names to trial ID will be kept and password-protected.

### Statistical methods

We intend to interview all randomized individuals even if they drop out of treatment permitting straightforward ITT analyses. Missing data will be addressed using full information maximum likelihood (FIML) methods [121]. We expect that missing data are minimized by using electronic data capture systems that enable real-time data monitoring. We will test for attrition bias by comparing baseline scores for those who are lost to follow-up with those who complete follow-up. If analyses suggest missing data that violates missing at randomness, we will use Bayesian estimation or pattern modeling in place of FIML [122].

We will evaluate non-normality, variance heterogeneity, specification error, and outlier effects in all analyses. We generally will rely on robust methods of analysis (e.g., Huber-White robust standard errors in Mplus or bootstrapping) [122]. We will make clustering adjustments as implemented in the Mplus software, as needed. For all multi-item measures, we will evaluate composite reliability [123], concurrent validity, and discriminant validity. We will routinely test for unidimensionality and explore the factor structure of all multi-item scales. These analyses will dictate the formation of latent variables to accommodate variable inter-correlations and collinearity, as necessary. We will adjust for familywise error rates using a Holm-modified Bonferroni method [122, 124, 125] and will compare results with unadjusted contrasts in the spirit of sensitivity analyses.

#### Data analysis: statistical power analysis

The field has generally operationalized a clinically meaningful effect as a Cohen’s *d* of 0.50 or a correlation equivalent to it of 0.23, the latter of which represents about 5% explained variance [126–128]. Rather than framing power analysis as the probability of correctly rejecting a null hypothesis, we approach it in terms of effect size sensitivity. If a minimal clinically meaningful effect size is set at *d* = 0.50, it does not matter if we “miss” effect sizes less than *d* = 0.50 by failing to reject the null hypothesis for them because they are judged to be non-meaningful [129, 130].

Assuming conservatively a 12% failure to assess individuals, our final sample size of complete case data will be about 211 or about 105 per condition. For a traditional single degree of freedom contrast of means to evaluate paths from treatment to mediator and assuming a power of 0.80, the effect size sensitivity for a sample size of 100 per group is a population Cohen’s *d* of 0.39, which represents about 3.5% explained variance. This sensitivity will decrease somewhat if there is clustering, but the decrease will be offset by the use of baseline covariates. For a regression analysis to estimate paths from mediator to outcome with 8 predictors (to account for the simultaneous entry of mediators and covariates to control), and assuming a squared multiple correlation of 0.40, the effect size sensitivity for a given predictor will be the detection of a population coefficient that represents 3% unique explained variance. If we add a cluster adjustment representing an interclass correlation coefficient of 0.10, the sensitivity increases to 5% unique explained variance. These same statements apply for the analysis of moderation. The full information estimation structural equation model (SEM) we reference below will generally have equivalent if not greater statistical power than the above, so they, by definition, are adequately powered [131].

#### Data analysis: analysis of aims

**Fig. 2:**
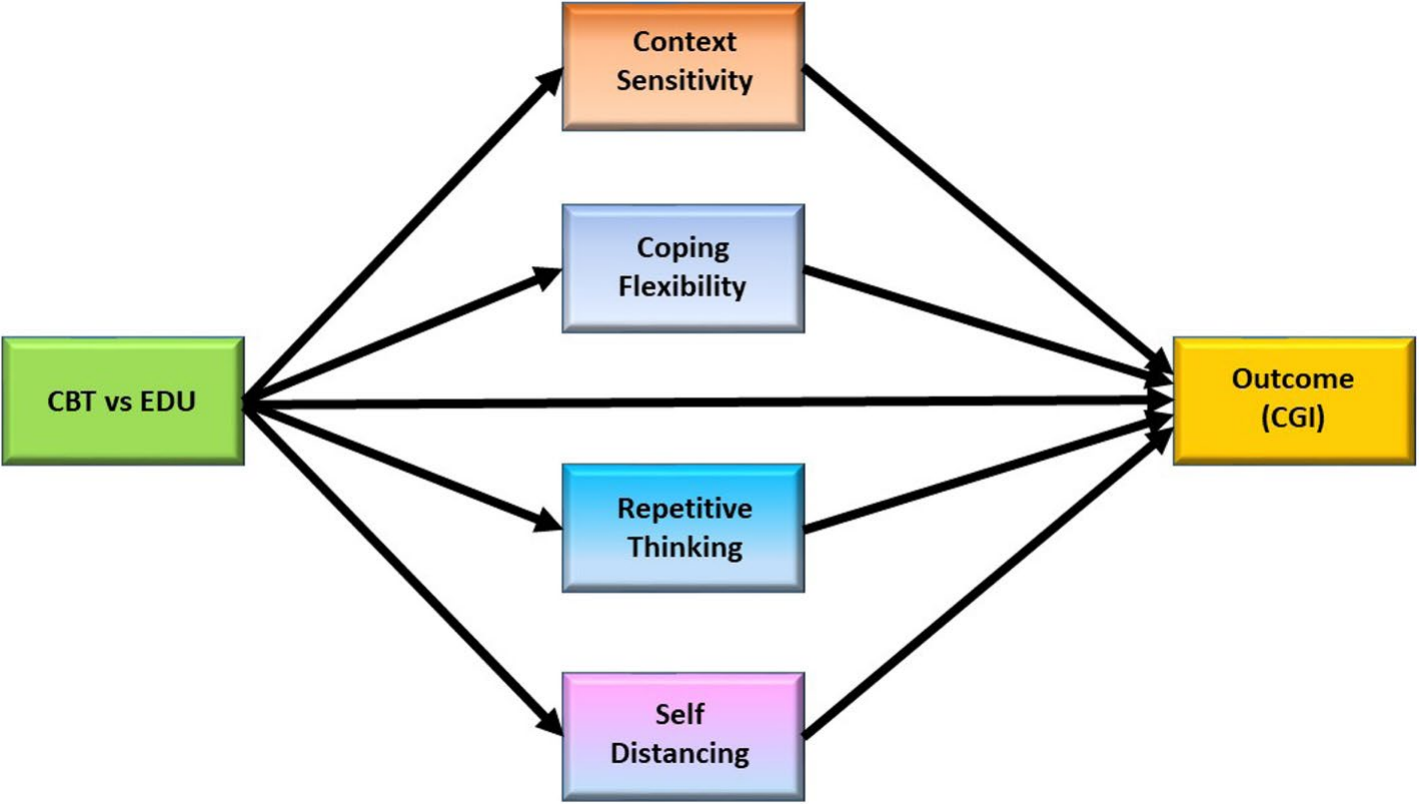
Randomized experimental trial model of mediation analyses

Given the centrality of mediation and moderation in this proposal, we will use analytic methods based in SEM for RCTs [132]. We refer to our approach as a randomized explanatory trial (RET) because of its emphasis on the *explanation* of treatment effects on outcomes through mediation and moderation. The exogenous treatment condition is identified by a two-valued variable (0 = EDU, 1 = CBT). The endogenous outcome, our primary endpoint, is the CGI. Mediators are the primary transdiagnostic change mechanisms of the intervention. A powerful feature of RET analysis is that it pinpoints the specific facets where a program succeeds and where it falls short of eliciting mechanistic change that drives symptom relief. RET is not concerned so much with omnibus mediation effects but rather focuses on each link in a given mediational chain and identifies the link(s) where a chain is “broken” so that we learn what needs to be addressed technically to boost the therapeutic impact of CBT. See Fig. 2 for the RET schematic, which will be analyzed overall using SEM.

Also of interest in an RET is the significance and magnitude of paths linking each mediator to the outcome. For example, perhaps a path associated with coping flexibility is not significant, suggesting that, contrary to our assumptions, coping flexibility does not meaningfully elicit UCPPS symptom improvement. In this case, we might consider streamlining the program so that it is simpler by eliminating the flexible problem solving. We will conduct state-of-the-art dominance analyses that allow us to order the relative strength of paths from the mediators to the outcome variable [133].

*Analyses for Aim 1:* Evaluate the efficacy of MC-CBT for UCPPS as compared to a nonspecific control intervention (EDU) in relieving pain and related symptoms. *H1a* states: Patients randomized to MC-CBT will show greater global symptom improvement on primary endpoint (CGI) compared to those randomized to EDU. This hypothesis will be tested using a covariate-adjusted single degree of freedom contrasts at each time point (immediate posttest 2 weeks, 3 and 6 months) comparing MC-CBT versus EDU on the full-scale CGI. Standard covariates will include medication history, gender, age, and indicators of symptom severity as measured at baseline. *H1b* states: MC-CBT will be superior to EDU on key secondary endpoints including the severity of pelvic pain and urinary symptoms, and functional impairment at post treatment. The same analytic strategy for H1a will be applied to secondary endpoints. For secondary outcomes, available baseline data will be included as a covariate.

*Analyses for Aim 2:* Evaluate the durability of MC-CBT relative to EDU at 3 months and 6 months following treatment discontinuation. This will be tested using the same approach as Aim 1 that is focused on the immediate posttest but now it will be focused on the time points of 3 months and 6 months, separately. The group differences in effects at one time point can be compared to the group difference at another time point using a single degree of freedom interaction contrast with time as a within-subject moderator, using a robust estimator (e.g., Huber-White or bootstrapping).

*Analyses for Aim 3:* Identify theoretically relevant change mechanisms that explain how and for whom transdiagnostically designed MC-CBT is more effective for the purpose of optimizing treatment effects. *H3a:* To evaluate the mediational dynamics that account for the precise cognitive processes that explain CBT’s effectiveness, we will focus on two types of analyses: (a) limited information estimation [134, 135] that focuses on selected mediational paths at a given time point (with exceptions noted below, where we introduce time lags), and (b) more complex mediational modeling using variants of full information SEM. For the former, we will first compare mean scores on each mediator for the MC-CBT versus EDU conditions using a robust single degree of freedom contrasts and using the baseline measure as a covariate and other covariates per Aim 1. These analyses provide limited information estimates of paths for each mediator at each time point. This between-group, ANCOVA-based framework is superior to more traditional mixed modeling strategies [136].

A second set of limited information estimation analyses will be performed that regress a given outcome (e.g., CGI) onto one or more variables from each of the transdiagnostic mediators as well as the treatment condition dummy variable and selected covariates (see Fig. 2). Importantly, given we will assess mediators at multiple time points during treatment as well as at the posttest and after, we also will be able to estimate effects of the mediator at time t-1 on the outcome at time t, thereby taking into account sequencing of time intervals. These analyses must be strategic and conducted relative to operative time dynamics.

*H3b: Trait self-regulation will moderate the impact of MC-CBT versus EDU on UCPPS symptom improvement both post treatment and at follow-up; those with higher baseline self-regulation will benefit more from MC-CBT than those with lower self-regulation.* This hypothesis posits the existence of moderated effects in the broader RET framework. A strength of RET analysis is that it can pinpoint where in the broader causal framework the moderated effect occurs. There are two loci where moderation might occur. First, low trait self-regulation might disrupt the effect of the treatment on the mediator; individuals low in self-regulation may lack the cognitive resources to complete the cognitive change tasks demanded of CBT and, as a result, the strength of path *from treatment to mediator* will be lower for low self-regulators than for high self-regulators. Another possibility is that those with low trait self-regulation cannot translate the skills they learn in coping skills training (e.g., flexible problem solving) to ease the burden of painful UCPPS symptoms, hence it disrupts the path *between moderator and outcome*. A powerful feature of RET analysis is that instead of merely identifying behavioral self-regulation as a treatment-outcome moderator, we identify where in the mediational chain it is impacting treatment effects. These tests will be executed using product term analyses with robust standard errors in SEM/regression contexts.

### Interim analyses

Because of the safety profile of treatments, no interim analyses are planned.

### Oversight and monitoring

#### Data and safety monitoring

The EPPIC’s scientific integrity and safety will be monitored by an independent Data and Safety Monitoring Board (DSMB) composed of a urologist, biostatistician, and two behavioral scientists with subject matter expertise in chronic pain and transdiagnostic processes. The DSMB will meet annually. We will also appoint a Safety Officer who will serve as an independent evaluator (external to the study) of all adverse events (AEs), both serious and non-serious. In the case of this unmasked trial, the Safety Officer will work with the investigators to assure that any adverse event is fully documented. The Safety Officer will also review adverse event data to assess if the frequency of the AEs changes dramatically from baseline. Study-related adverse events will be reported to the NIDDK, Data Safety Monitoring Board (DSMB), and UB Institutional Review Board annually.

#### Consent and protection from risks

All participants deemed eligible will be consented during their baseline visit to the clinic. Consents will be conducted by a therapist or graduate research assistant. Each participant will be asked to sign the consent form and provided a copy. The consent form will provide an explanation of the purpose of the research, the expected duration of the individual’s participation, a description of the procedures, description of any reasonably foreseeable risks and benefits, a statement ensuring confidentiality and the voluntary nature of participation, information on compensation and intervention available, and information regarding who to contact for information on the research and their rights as a research participant. Risk reduction will include the preparation of a proactive policy for monitoring, assessing, and responding to suicide risk. During the acute phase, any participant showing significant deterioration or developing active suicidal potential as judged clinically by the treating therapist with confirmation of testing data will be removed from the structured protocol and triaged immediately to more intensive clinical intervention in the community. The policy is the product of a larger effort that emphasizes best practices for managing individuals at increased risk for suicide in clinical research setting by training and supervising staff to identify suicide risk in study patients, identifying community resources to respond to risk, and documenting and reporting suicidal behaviors.

### Adverse events and harms

Study-related adverse events will be reported to the NIDDK, EPPIC DSMB, and University at Buffalo Human Subjects Institutional Review Board (IRB) annually. Serious adverse events (death, life-threatening, new, serious, or permanent disability) that are determined to be related to the study procedures will be reported to the UB IRB within 72 h and will be reported to the NIDDK Program Officer within 7 days of incident. Other serious, unexpected, and treatment-related adverse events will be reported to the NIDDK Program Official within 15 days and to the UB IRB within ten business days. The proposed trial will be stopped only if the DSMB believes there is an unacceptable risk of serious adverse events attributable to one of the treatment arms. In this case, the DSMB could decide to terminate one of the arms of the trial or the entire trial.

#### Confidentiality

Confidentiality and the protection of protected health information will be ensured by following the Privacy Rule of the Health Insurance Portability and Accountability Act (HIPAA) guidelines and the regulatory guidelines for these issues as required by the UB Institutional Review Board. All study participants will be assigned a unique ID number that will be linked to their contact information through an encrypted and password-protected electronic file stored securely and separately from their research data. Digital files will be maintained in a computerized database housed on a username and password-protected fileserver. Hard copy and digital data will only be accessible by authorized study personnel formally trained in research ethics and compliance. All EPPIC personnel are required to complete human subjects protection training through the Collaborative Institutional Training Initiative (CITI) program. We further protect the confidentiality and privacy of participants using several data safety and protection safeguards of the study using REDCap which restricts data entry rights and access to team members with data management roles and responsibilities, logs all user activity, and builds an audit trail within the project.

#### Plans for auditing trial conduct

We do not anticipate EPPIC will require external auditing as internal controls (e.g., IRB, DSMB) is sufficient for enduring appropriate oversight and monitoring. In addition, EPPIC will incorporate multiple oversight mechanisms our team used in similar NIH trials and independently validated through rigorous external auditing for minimizing risk of protocol violations (i.e., serious divergence from the protocol that materially (a) reduces the quality or completeness of the data, (b) makes the Informed Consent Form inaccurate, or (c) impacts a subject's safety, rights, or welfare) including:

- Inadequate, absent, or delinquent informed consent
- Inclusion/exclusion criteria not met
- Unreported serious adverse events
- Use of prohibited therapies
- Incorrect or missing testing
- Multiple visits missed or outside permissible windows
- Materially inadequate record keeping
- Intentional deviation from protocol, Good Clinical Practice, or regulations by study personnel
- Subject repeated non-compliance with study requirements

#### Plans for communicating protocol amendments to relevant parties

Any substantive amendments to the study protocol would require approval by the UB IRB. Once approved, they would then be reported in the trial registry. Any amendment will be reported in the final report of study findings.

### Organizational structure

**Fig. 3:**
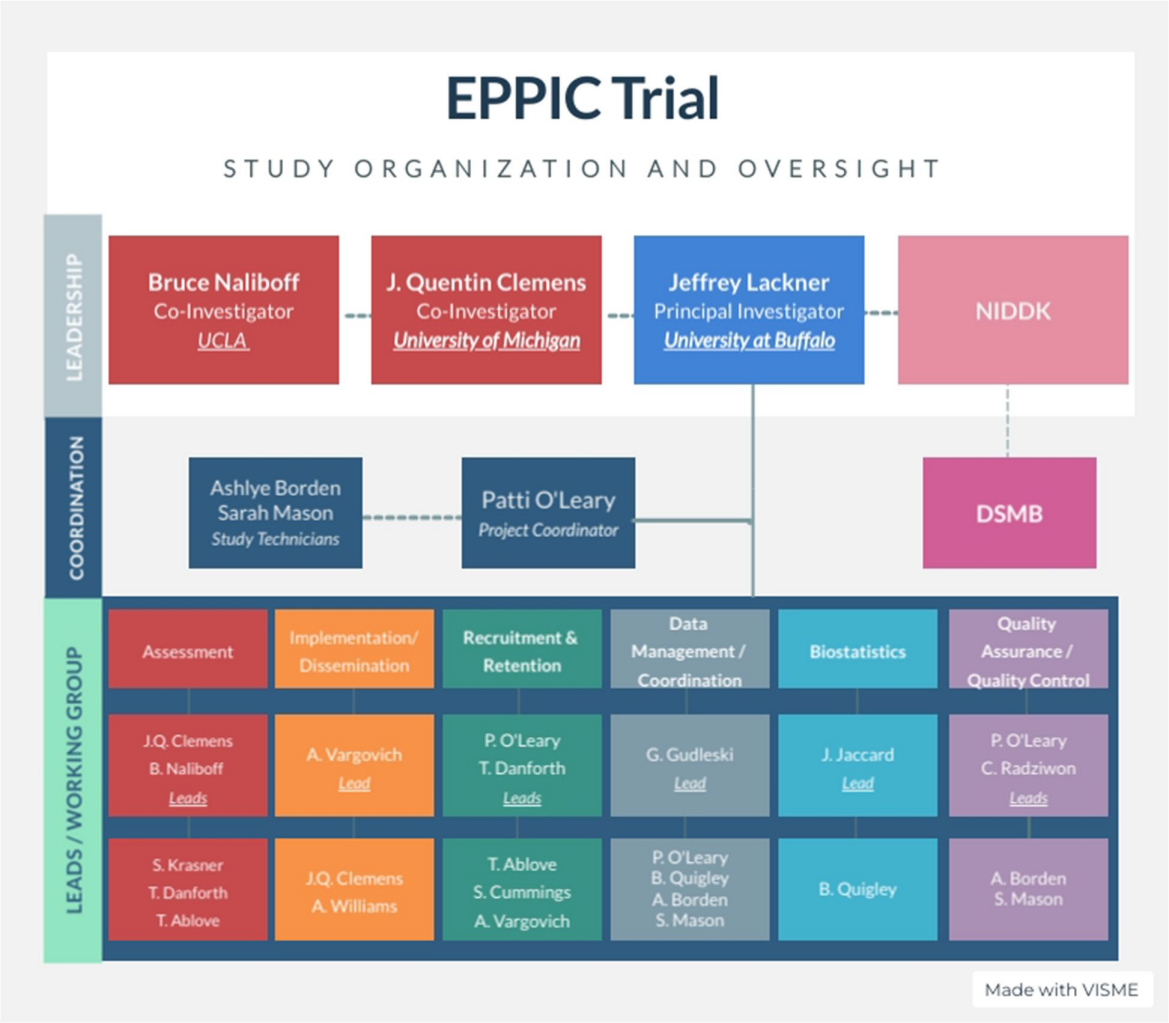
EPPIC organizational structure and key personnel

The proposed study will be led by Dr. Jeffrey Lackner who as Project PI will provide overall scientific and administrative oversight, leadership, and guidance for the project. Co-investigators and research staff will be organized into six focus teams: Assessment, Implementation/Dissemination, Recruitment/Retention, Data Management/Coordination, Quality/Assurance, and Statistics (Fig. 3).

### Dissemination policy

The main goals guiding the dissemination of findings are (1) to inform public perception, understanding, and awareness of UCPPS; (2) to expand our understanding of the underlying mechanisms of UCPPS symptoms as a public health challenge; (3) to increase adoption of new developed evidence-based interventions UCPPS patients with treatment-resistant symptoms; and (4) to contribute productively to positive and innovative changes in health care policy. By registering the trial with ClinicalTrials.gov and submitting results in a timely fashion, findings will be readily available to the public and professionals alike. Results will be shared through traditional scientific avenues, such as conference presentations and peer-reviewed journal articles in urology, pain, and behavioral medicine/behavior therapy. All publications resulting from this project will be deposited to PubMed. Beyond the medical and allied health communities, we will prepare material for publication in local and national health and lifestyle periodicals and websites. For all manuscripts, we will subscribe to the most common and frequently referenced authorship guidelines in biomedicine issued by the International Committee of Medical Journal Editors (ICMJE). It is not our practice to use professional writers in the preparation of manuscripts. Dissemination efforts will be strengthened by the office of UB Media Relations which aids in the sharing of research findings to news sources through frequent electric newsletters as well as direct press releases written by the media relations department staff in collaboration with the researcher. We will consult face to face with health care policy makers and with health-related media experts who have supported our work to expand the reach and real-world applicability of research findings emerging from this project. We will collaborate with patient advocacy groups (e.g., Interstitial Cystitis Association) and professional organizations (American Psychological Association, Society for Behavioral Medicine, International Association for Study of Pain, American Urological Association, Society of Urodynamics, American Academy of Pain Medicine, Association for Psychological Science, etc.) to disseminate summaries of the primary results to members and other stakeholders, including patients, policy makers, payers, and health care system partners as we have done with prior trials. We will provide lay language summaries of research findings to community partners (e.g., pharmacies, supermarkets, managed care organizations) who supported recruitment efforts and prioritize self-management as a strategic corporate goal and we will take advantage of social media outlets to publicize our findings.

## Discussion

Clinical trials, particularly behavioral pain ones, have rapidly evolved over the past 10 years. “Horse race” efficacy questions that singularly focus on whether Treatment X works better than Treatment Y are insufficient for understanding clinical outcomes of multisymptom disorders among heterogeneous patients. That these disorders are targeted through treatments that vary by procedure and provider and subject to the impact of both technical and nonspecific processes (e.g., treatment expectancy, therapeutic alliance) at different phases of treatment adds multiples layers of complexity to the penultimate goal of establishing an efficacy profile of novel therapy in the context of a randomized clinical trial. This means that contemporary trials must not only shed light on whether a treatment works but the mechanisms responsible for effects, their timing, the subtypes of patients for whom treatment is most effective, and their enduring benefit after therapy terminates. Drawing upon the state-of-the-art trial architecture of the IBS Outcome Study [137], EPPIC has been designed to systematically address these questions to optimize scientific integrity and practice-changing potential.

One novel feature of EPPIC is our RET analytic approach for establishing whether the specific technical components that comprise transdiagnostic CBT for UCPPS work for the reasons previously hypothesized. RET allows us to unpack the specific strategies responsible for therapeutic benefit. If we isolate a specific therapeutic technique that impacts symptom relief by, for example, increasing cognitive flexibility, that strategy (e.g., flexible problem solving) can be isolated, accentuated, or amplified. Because symptom improvement is “time stamped” through repeated assessments in outcome and mediator when the corresponding module is introduced, clinical researchers may use information emerging from RET to re-sequence a particularly robust intervention if benefit occurs earlier than originally, thereby reducing dropout and maximizing patient satisfaction and benefit. If we find that a treatment module is relatively inert (i.e., introduction of a specific strategy does not correspond with a change in corresponding mediator or symptoms), then it can be abbreviated, modified, or jettisoned altogether. A microanalytic RET approach represents a major methodological innovation that can help engineer low-intensity treatments that are more efficient, less complex, and easier to disseminate and implement in routine clinical practice where uptake of evidence-based protocols is low.

We also improve on other behavioral pain trials by exploring prescriptive and prognostic factors that impact treatment outcomes. Each provides different information about how a pretreatment variable can inform the predictive relationship with outcome. *Prescriptive* variables (i.e., moderator) predict a different pattern of outcomes between two or more treatments*,* whereas *prognostic* variables predict outcomes independent of treatment. Because they are general and non-specific, prognostic variables can specify which types of patients respond favorably to treatment regardless of which treatment they receive. Patients with a specific prognostic baseline profile may require tighter oversight to achieve treatment gains. Prescriptive variables have more fine-grained clinical value because they shed light on who will do best in which treatment. The literature often obscures these distinctions, but clarity will be increasingly important in the age of personalized medicine when resources are limited and it is increasingly important to develop clinical decision algorithms that identify types of patients who will do best in certain treatments at minimal economic and personal cost. By using an active credible education control, we address a criticism against behavioral pain trials that have relied on passive wait list controls to establish their efficacy. Passive controls do “not provide most of the variables which occur within a psychotherapeutic process” [138], making it difficult to know whether effects ascribed to CBT are due to factors unique to it (e.g., remediation of cognitive skills) or nonspecific ones (e.g., treatment expectancy) that are part of any given treatment [139]. Additional EPPIC strengths include randomization of participants to two manualized, conceptually and technically distinct treatments identical in format, frequency, duration, home exercises, and level of therapist training; use of “blind” assessors to validate patient-reported outcomes; a psychometrically sound assessment battery; well-defined eligibility criteria that balance internal and external validity; a well-articulated missing data plan; and delayed post baseline assessment that can isolate effects of unobscured by participants involvement in therapy as happens when post treatment assessment occurs at the end of the acute phase (e.g., week 10 of 10-week acute phase) but before treatment is discontinued. By subscribing to IMMPACT recommendations for clinical pain trials [140, 141], outcome assessment is sufficiently broad to characterize a range of treatment effects beyond pain relief. Self-selection is a potential source of bias that threatens the external validity of RCTs if recruitment results in a selective sample that distorts true treatment effects with limited generalizability. EPPIC will minimize this bias by implementing a diversified, proactive recruitment plan that seeks to generate a broad spectrum of participants reflecting “the entire eligible patient population” (symptomatic community and clinic patients) yielding a less biased efficacy profile (tertiary care patients represented in most behavioral pain trials are more responsive to psychosocial pain therapies than symptomatic community ones [142]), that promotes generalizability and facilitates the translation of trial results into real-world practice [143].

A relative limitation of the EPPIC is the absence of a biological marker that would shed light on physiological mechanisms responsible for CBT-induced symptom relief. The EPPIC will be conducted at one site which may limit the generalizability of study findings to other treatment locations and populations. We would prefer to assess the durability of treatment effects over 12 months but that is not feasible here. Nor will we conduct cost utility assessments as we have done with other MC-CBT trials. We do not expect the cost of MC-CBT for UCPPS to differ significantly from the MC-CBT regimen featured in our previous work [52] and therefore do not believe cost utility analyses are sufficiently warranted. While a self-guided digital version of our CBT protocol is potentially more scalable, digital therapeutics without therapist contact are vulnerable to low persistence undermining their value proposition [144].

## Conclusions

The EPPIC study will test the efficacy of a largely home-based version of CBT transdiagnostic in scope for UCPPS with reference to a non-specific support education and identify the patients for whom it is most effective and the theory-based change mechanisms that drive UCPPS symptom improvement (e.g., pelvic pain, urinary symptoms). An effective, brief, low-intensity, and relatively simple “across disorder” or transdiagnostic treatment that simultaneously targets what centralized pain disorders such as UCPPS have in common rather than their differences would improve on conventional behavioral pain approaches whose narrower focus, length, cost and/or complexity have limited their transfer from well-controlled research to “real world” settings accessible to more clinicians and individuals in need. By capitalizing on cutting-edge cognitive science research that emphasizes the importance of how patients think (e.g., cognitive flexibility) and informs self-regulatory capacity — not simply what they think about as, for example, pain beliefs like pain catastrophizing (the focus of traditional behavioral pain treatments) — we plan to demonstrate how a parsimonious, transdiagnostic, mechanistically-driven behavioral treatment of low intensity can reduce both symptom burden of UCPPS with minimal clinician oversight and maximum patient engagement, convenience, safety, and benefit.

## Supplementary Information

**Additional file 1.**

## Data Availability

After the investigators complete analyses and publication of study aims, deidentified data, statistical code, study protocol, and other project materials will be prepared for researchers who wish to collaborate on specific analyses as requested and to investigators who are conducting meta-analyses. All public requests for such materials will be evaluated by the PI and decisions at his discretion will be made on an individual basis with the goal of disseminating study findings to a broad network of stakeholders.

## Abbreviations

AUA: American Urological Association
BRIEF-A: Behavior Rating Inventory of Executive Functioning – Adult
BPS: Bladder pain syndrome
BSI-18: The Brief Symptom Inventory – 18
CFS: Chronic fatigue syndrome
COPC: Chronic Overlapping Pain Condition
CPP: Chronic pelvis pain
CP/CPPS: Chronic prostatitis/chronic pelvic pain syndrome
CSQ: Client Satisfaction Questionnaire
CGI: Clinical Global Improvement
CMSI: Complex Multi-Symptom Inventory
CBT: cognitive behavioral therapy
CITI: Collaborative Institutional Training Initiative
CSI: Context Sensitivity Index
CFS-R: The Coping Flexibility Scale – Revised
DSMB: Data and Safety Monitoring Board
EPPIC: Easing Pelvic Pain Interventions Clinical Research Program
EDU: Education condition
ES: Effect size
EQ-D: Experiences Questionnaire – Decentering
FM: Fibromyalgia
FIML: Full information maximum likelihood
GERD: Gastroesophageal reflux disease
GUPI: Genitourinary Pain Index
HIPAA: Health Insurance Portability and Accountability Act
ITT: Intent-to-treat
ICMJE: International Committee of Medical Journal Editors
IC: Interstitial cystitis
ICPI: Interstitial Cystitis Problem Index
ICSI: Interstitial Cystitis Symptom Index
IBS: Irritable bowel syndrome
MC: Minimal contact
MAIA-2: Multidimensional Assessment of Interoceptive Awareness – 2
OB/GYN: Obstetrician-Gynecologists
PPI SF-6a: Pain interference
PTQ: Perseverative Thinking Questionnaire
CERQ-PB: The Perspective Broadening scale of the Cognitive Emotion Regulation Questionnaire
QOL: Quality of life
RCT: Randomized clinical trial
RET: Randomized Explanatory Trial
RWN: Reflective writing narrative
RNT: Repetitive negative thought
REDCap: Research Electronic Data Capture
SEM: Structural Equation Modeling
TMD: Temporomandibular joint disorder
UCPPS: Urologic chronic pelvic pain syndrome
